# Acute lymphoblastic leukemia necessitates GSH-dependent ferroptosis defenses to overcome *FSP1*-epigenetic silencing

**DOI:** 10.1016/j.redox.2022.102408

**Published:** 2022-07-31

**Authors:** Lucas B. Pontel, Alberto Bueno-Costa, Agustín E. Morellato, Juliana Carvalho Santos, Gaël Roué, Manel Esteller

**Affiliations:** a Cancer Epigenetics Group, Josep Carreras Leukaemia Research Institute (IJC), Badalona, Barcelona, Catalonia, Spain; b Instituto de Investigación en Biomedicina de Buenos Aires (IBioBA), CONICET-Partner Institute of the Max Planck Society, Buenos Aires, Argentina; c Lymphoma Translational Group, Josep Carreras Leukaemia Research Institute (IJC), Badalona, Barcelona, Catalonia, Spain; d Centro de Investigacion Biomédica en Red de Cáncer (CIBERONC), Madrid, Spain; e Institucio Catalana de Recerca i Estudis Avançats (ICREA), Barcelona, Catalonia, Spain; f Physiological Sciences Department, School of Medicine and Health Sciences, University of Barcelona (UB), Barcelona, Catalonia, Spain

**Keywords:** Glutathione, Ferroptosis, Acute lymphoblastic leukemia, GPX4, FSP1, DNA Methylation

## Abstract

Ferroptosis is a form of cell death triggered by phospholipid hydroperoxides (PLOOH) generated from the iron-dependent oxidation of polyunsaturated fatty acids (PUFAs). To prevent ferroptosis, cells rely on the antioxidant glutathione (GSH), which serves as cofactor of the glutathione peroxidase 4 (GPX4) for the neutralization of PLOOHs. Some cancer cells can also limit ferroptosis through a GSH-independent axis, centered mainly on the ferroptosis suppressor protein 1 (FSP1). The significance of these two anti-ferroptosis pathways is still poorly understood in cancers from hematopoietic origin. Here, we report that blood-derived cancer cells are selectively sensitive to compounds that block the GSH-dependent anti-ferroptosis axis. In T- and B- acute lymphoblastic leukemia (ALL) cell lines and patient biopsies, the promoter of the gene coding for FSP1 is hypermethylated, silencing the expression of *FSP1* and creating a selective dependency on GSH-centered anti-ferroptosis defenses. *In-trans* expression of FSP1 increases the resistance of leukemic cells to compounds targeting the GSH-dependent anti-ferroptosis pathway. FSP1 over-expression also favors ALL-tumor growth in an *in vivo* chick chorioallantoic membrane (CAM) model. Hence, our results reveal a metabolic vulnerability of ALL that might be of therapeutic interest.

## Highlights

• Hematopoietic-derived cancer cell lines depend on GSH metabolism.
• Acute lymphoblastic leukemia (ALL) cell lines are sensitive to ferroptosis inducers.
• Ferroptosis suppressor protein 1 (FSP1) is silenced by DNA methylation in ALL.
• Ferroptosis inducers might be a selective therapeutic option for ALL patients.

## Graphical abstract

**Figure:**
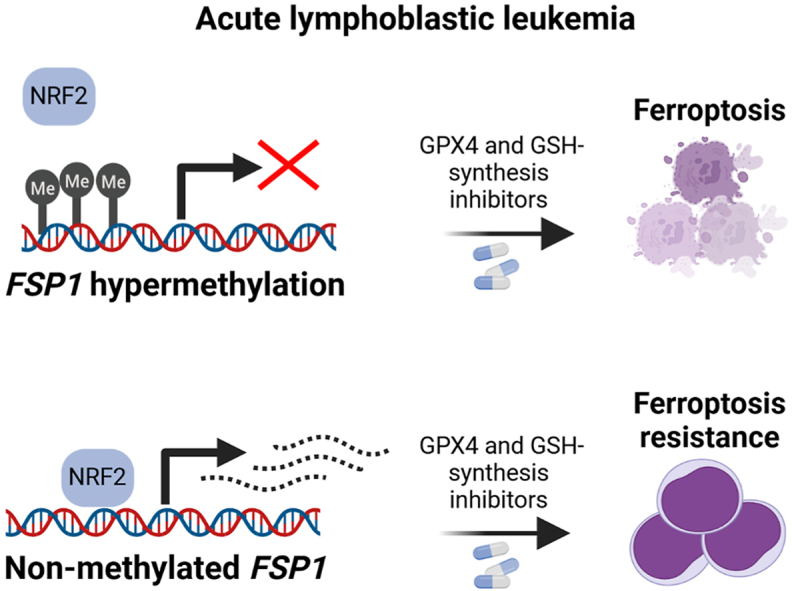

## 1 Introduction

**Fig. 1:**
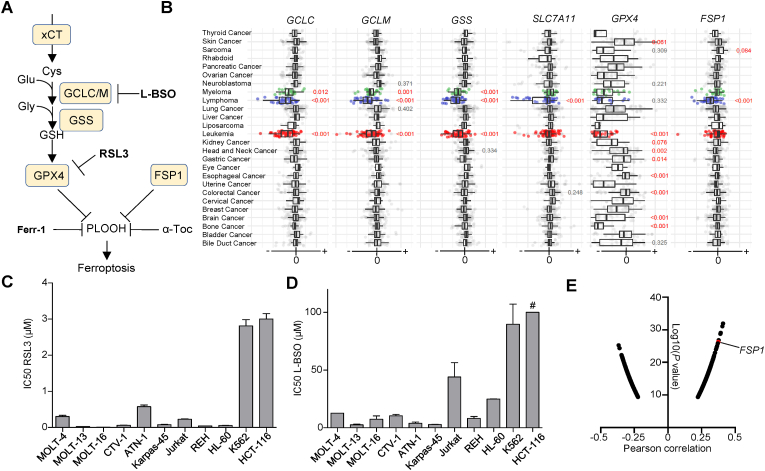
**GSH metabolism is a vulnerability in acute lymphoblastic leukemia (ALL) cell lines. A.** Scheme depicting glutathione (GSH) metabolism and the main cellular ferroptosis defenses. **B.** Cancer dependency maps for *SLC7A11*, *GCLC*, *GCLM*, *GSS*, *GPX4* and *FSP1.* The statistical analysis was performed applying a one-way ANOVA and a Tukey test for multiple comparison. **C.** IC50 (Inhibitory concentration at 50%) of 1*S*,3*S*-RSL3 (RSL3) for the cell lines enumerated in the x-axis. The IC50 was calculated from viability curves shown in Sup. Fig. 1A (mean ± SD, n = 3). **D.** IC50 of l-buthionine-sulfoximine (L-BSO) from viability curves shown in Sup. Fig. 1B (mean ± SD, n = 3). # indicates that for HCT-116 the IC50 was >100 μmol L^−1^ **E.** Log10(*P* value) of the Person correlation between area under the curve (AUC) toxicity data for RSL3 and gene expression in all the cell lines with available data at depmap.org. *FSP1* ranks 6th among the genes with positive correlation, suggesting high *FSP1* expression is usually accompanied by high resistance to RSL3. xCT (SLC7A11/SLC3A2), GCLC/GLCM (Gamma glutamyl-cysteine ligase), GSS (GSH synthase), GPX4 (GSH peroxidase 4), FSP1 (Ferroptosis suppressor protein 1), α-Toc (α-tocopherol), μM (μmol L^−1^).

Cells can undergo death through several mechanisms such as apoptosis and necroptosis [1]. In the last decade, an iron-dependent form of cell death was described termed ferroptosis. This mechanism is independent of the classical apoptosis pathway, and it is characterized by the accumulation of phospholipid hydroperoxides (PLOOH), inflicting lethal damage to cell membranes [2]. *In vitro*, ferroptosis can be reverted by iron chelators or by PLOOH-specific antioxidants such as ferrostatin-1 (Ferr-1) and vitamin E (α-tocopherol) [[3], [4], [5]]. To prevent this form of cell death, cells evolved the protein glutathione peroxidase 4 (GPX4), which metabolizes PLOOH using glutathione (GSH) as cofactor [6] (Fig. 1A). The genetic inactivation or the chemical inhibition of GPX4 by compounds such as (1*S*,3*R*)-RSL3 (RSL3) trigger cell death through ferroptosis [7]. Blocking GSH synthesis by the compound l-buthionine sulfoximine (L-BSO) also triggers ferroptosis, though this effect largely depends on the cell line [8]. GSH is synthesized from cysteine and glutamate by the enzyme glutamate cysteine ligase (GCL), which is the target of L-BSO and it is formed by a catalytic (GCLC) and a regulatory (GCLM) unit [9]. Cysteine, for GSH synthesis, can be obtained from the metabolization of cystine, which is imported through xCT (SLC7A11/SLC3A2). Accordingly, inhibition of xCT by erastin or sulfasalazine also triggers ferroptosis [2]. The last step of GSH synthesis is the addition of glycine carried out by the enzyme glutathione synthase (GSS).

Several reports have described mechanisms that prevent ferroptosis independently of GSH. The main factor associated to this GSH-independent ferroptosis defense is known as ferroptosis suppressor protein 1 (FSP1) [[10], [11], [12]]. Initially, FSP1 was named as AIFM2 (Apoptosis inducing factor mitochondrial 2) based on sequence homology to apoptosis factor mitochondrial-associated 1 (AIFM1), and later renamed as FSP1 [13]. This factor presents NADH:ubiquinone oxidoreductase activity and it has been shown to limit ferroptosis by regenerating the coenzyme Q_10_ [11]. This ubiquinone appears as a lipid-soluble antioxidant and might catalyze the detoxification of PLOOHs [14]. The interplay between FSP1 and GSH-dependent ferroptosis defenses has been addressed in several systems, including xenograft models of lung cancer [10]. However, it is less understood the role of FSP1 in hematopoietic cancers. Here, we show that FSP1 is barely expressed in ALL cell lines. This lack of expression correlates with the DNA hypermethylation of *FSP1* promoter in cells and patient biopsies and creates a selective dependency of ALL cells on GSH-dependent ferroptosis defenses.

## 2 Material and methods

### 2.1 Cell lines

Jurkat, CTV-1, MOLT-4, MOLT-13, MOLT-16, SUPT-1, Nalm6, REH, Kasumi, Karpas-45, K562, ATN-1 and HL-60 were grown in Roswell Park Memorial Institute 1640 medium (RPMI-1640) supplemented with 10% fetal bovine serum (FBS) and 1% penicillin/streptomycin. HCT-116 and HEK293T cells were maintained in Dulbecco's Modifies Eagle's Medium (DMEM), high glucose (4.5 g/L), supplemented with 1% penicillin/streptomycin and 10% FBS. Unless otherwise stated, cell lines were obtained from American Type Culture Collection (ATCC). All cell lines were authenticated by short tandem repeat profiling (LGS Standards) and tested negative for mycoplasma infection.

### 2.2 Patient data

All the patient-derived data shown in this manuscript were obtained from anonymized open databases. Specifically, the CpG promoter methylation data were obtained mining primary tumors from hematopoietic and lymphoid origin available at The Cancer Genome Atlas (TCGA) (https://www.cancer.gov/about-nci/organization/ccg/research/structural-genomics/tcga) and from published datasets generated by Esteller's lab [[15], [16], [17]]. CpG methylation from normal donors was obtained from the previous works [[18], [19], [20], [21]]. A total of 1259 tumor biopsies and normal samples from hematopoietic and lymphoid compartment were analyzed (data shown in Fig. 5a). Survival data were obtained from TCGA and TARGET cohorts through the platform cBioPortal. Gepia2 was used to analyze survival data across the 32 TCGA cohorts [22].

### 2.3 Viability assays

Viability was determined by resazurin dye (Sigma-Aldrich, #R7017). Briefly, 3000 cells per well were seeded into 96-well plate at a final volume of 200 μL. Depending on the cell line, at day 4 or 6 resazurin was added to a final concentration of 30 μmol L^−1^, and plates were left in the incubator for 3–5 h. Fluorescence (λ_**ex**_ = 525 nm; λ_em_ = 590 nm) was measured 3 h later and viability calculated as a percentage relative to the untreated well. Every experiment was performed by technical triplicate and repeated at least 3 times. Reagents used were: 1S,3S-(RSL3) (MedChem Express, #HY-100218A), l-buthionine sulfoximine L-BSO (Sigma, #B2515), Ferrostatin-1 (MedChem Express, # HY-100579), Necrostatin-1 (MedChem Express, # HY-15760), quinoline-Val-Asp-Difluorophenoxymethylketone (QVD) (MedChem Express, # HY-12305), *tert*-butyl hydroquinone tBHQ (MedChem Express, #HY-100489), 5′-aza-2′-deoxycytidine (DEC) (Sigma, # A3656). Fluorescence was determined in a Synergy H1 microplate reader (Biotek).

### 2.4 Flow cytometry

Independent cultures were set in 24-well plates, grown in presence of the indicated condition for 24 h, and then stained with C11-BODIPY 581/591 (Thermofisher #D3861) at a final concentration of 5 μmol L^−1^. Stained cells were transferred to a 96-well plate and fluorescence intensity (FI) was determined using phycoerythrin (PE, λ_**ex**_ = 488 λ_**em**_ **=** 585 nm) and fluorescein isothiocyanate (FITC, excitation/emission of 488/530 nm) channels in a FACS-BD Canto II flow cytometer with a high throughput (HTS) adaptor. FlowJo v9 software (BD Biosciences) was used for analysis. The ratio between the geometric mean FI of PE and FITC was calculated and plotted relative to the untreated samples. C11-BODIPY shifts the fluorescence emission maximum from 590 nm to 510 nm when it is oxidized.

### 2.5 FSP1 in trans expression

For complementation of *FSP1* expression, the lentivirus construct pLOC-AIFM2 (FSP1, #PLOHS_100010824) was obtained from Horizon discovery. HEK293T cells were transfected with the vector expressing FSP1, psPAX2 (Addgene #12260) and pMD2. G (Addgene #12259) using JetPrime® Transfection Reagent (Polyplus #101000046). After 72 h, virus-containing medium was collected, filtered through a 0.45 μm membrane, and delivered into CTV-1 and Jurkat cells. Blasticidin at 4 μg/mL was used for selection, and GFP-positive cells were purified by two rounds of cell sorting, obtaining >90% of GFP + cells. Then, FSP1 expression was confirmed by western blot in the pool of sorted cells and using an anti-FLAG antibody. For generating the empty vector control, FSP1 was removed by restriction enzyme digestion from the pLOC-AIFM2 plasmid. Then, the empty vector was re-generated by using Gibson assembly, yielding the vector pLOC-EV.

### 2.6 Expression analysis

Quantitative reverse transcription PCR (qRT-PCR) was used to assess *FSP1* mRNA expression. Briefly, total RNA was extracted from cell pellets using the SimplyRNA kit (Promega #AS1340) in the automated Maxwell RSC device (Promega, #AS4500). 2 μg of total RNA were converted to cDNA using the RevertAid First Strand cDNA synthesis kit (Thermofisher Scientific, #K1622). For quantitative PCR, SYBR Green PCR Master Mix (Life technologies, #4312704) was used and *GAPDH* expression as housekeeping control. The primers used were: hGADPH Fwd 5′ TCAAGGCTGAGAACGGGAAG 3’; hGADPH Rv 5′ CGCCCCACTTGATTTTGGAG 3’; hFSP1 Fwd 5′ AGACGGACAAAGGCACAGAG 3’; hFSP1 Rv 5′ CAATGGCGTAGACGTTGCTG 3’.

Western blot was used to determine protein expression. Briefly, cell pellets were lysed in RIPA buffer (20 mM Tris-HCl pH 7.5, 150 mM NaCl, 1 mM EDTA, 1 mM EGTA, 1% NP-40), water-bath sonicated and lysed in Laemmli 1x sample buffer (62.5 mM Tris-HCl pH 6.8, 25% glycerol, 2% SDS, 0.01% bromophenol blue, 5% β-mercaptoethanol). Antibodies used were: anti-LaminB1 (Abcam, #ab16048, 1:2000); anti-GCLC (Santa Cruz Biotechnology, #sc-390811, 1:500); anti-GCLM (Santa Cruz Biotechnology, #sc-55585, 1:500); anti-FSP1 (Santa Cruz Biotechnology, #sc-377120, 1:500); anti-GPX4 (R&D Systems, #MAB5457, 1:1000); anti-β-tubulin-HRP (Abcam, #21058, 1:2000). Secondary antibodies were anti-mouse HRP conjugated-secondary antibody (Abcam, #ab9044, 1:5000) and anti-rabbit HRP conjugated-secondary antibody (Sigma, #A0545, 1:5000).

### 2.7 DNA methylation analyses

DNA methylation status at gene promoter was determined by DNA methylation microarrays and bisulfite genomic sequencing. For the methylation analyses across cell lines from different tissue origin (data shown in Fig. 4A), we used the Infinium Human Methylation BeadChip 450K (Illumina) [23]. The *in-silico* methylation analysis focused on the hematopoietic compartment was performed using the MethylationEPIC BeadChip 850K (Illumina) [24], and filtering the Sanger cell lines from hematopoietic and lymphoid origin [23]. For hematopoietic-derived patient and normal samples, the DNA methylation microarray used was the Infinium Human Methylation BeadChip 450K (Illumina). DNA quality checks, bisulfite modification, hybridization, data normalization, statistical filtering, β methylation were calculated after ssNoob normalization using *Minfi* R package (Bioconductor) as described previously [25]. Heatmaps were built using Next-Generation Clustered Heat Map Builder [26]. For bisulfite genomic sequencing, genomic DNA was converted using the EZ DNA Methylation-Gold Kit (ZYMO Research, #D5005). Bisulfite PCR products were transformed into competent bacteria and a minimum of 8 clones were sequenced to calculate methylation frequency. Bisulfite PCR primers for *FSP1* amplification used were Fwd 5′ GYGTGAGTTAGGTTTTTAATT 3′ and Rv 5′ AAAAACACTTTAAACCAAATCTAAA 3'. Results were analyzed with BioEdit software and methylated cytosines were mapped using a custom perl script (BSMapR).

### 2.8 Glutathione (GSH) determination

GSH was determined using GSH-Glo Glutathione Assay (#V6911, Promega). Briefly, cells were grown for 24 h in presence of RSL3 or L-BSO. Afterwards, 10000 live cells were transferred into a white 96-well plate, and the reagents applied following manufacturer instructions. Luminescence was recorded in a Synergy H1 microplate reader (Biotek). A GSH-standard curve was performed in parallel, and the content of GSH per cell in each sample calculated considering the 10000 cells seeded initially.

### 2.9 Chick embryo chorioallantoic membrane (CAM) model

Fertilized white Leghorn chicken eggs were purchased from Granja Santa Isabel, S. L. (Córdoba, Spain) and incubated for 9 days at 37 °C with 55% humidity. At day 9 of their embryonic development, eggs were cleaned with alcohol 70° and a window of an approximate 2 cm-diameter was drilled on top of the air chamber of the eggshell. Then, half million pEV or pFSP1 Jurkat cells were resuspended in 25 μL RPMI medium containing 10% FBS and 100 U/mL penicillin and streptomycin (Thermo Fisher Scientific, Inc.) and 25 μL Matrigel (Cultek, # 354234). The mix was incubated for 15 min at 37 °C and subsequently implanted into the CAM of each egg. The window was then covered with a sterile tape and the eggs were placed back in the incubator. At days 12 and 14 of their embryonic development, 0.5 μmol L^−1^ of RSL3 or vehicle (DMSO) diluted in RPMI medium were administered topically on the tumor-bearing CAMs. On the 16th day of development (7 days post-implantation), chick embryos were sacrificed by decapitation. Tumors were excised and carefully weighed to determine their mass.

### 2.10 Statistics

Spearman's correlation was used to address the association between expression and methylation data. Kaplan-Meier plots and Logrank (Mantel-Cox) tests were used to estimate overall survival (OS) through univariate and multivariate Cox proportional hazards regression models. Patients were stratified in those with expression higher or lower than the mean for the whole set. For TARGET T-ALL, we selected the samples containing *FSP1* expression determined by microarray. Statistical analyses (One-way ANOVA adjusted with a Tukey's test for multiple comparison) were carried out with GraphPad Prism 8. Comparison between two groups was carried out by unpaired *t*-test. Values of p < 0.05 were considered statistically significant. Specific details for statistical analysis can be found in the legend of each figure.

### 2.11 Datasets

All the data used were downloaded from *depmap. org* or cBioPortal and analyzed using R-environment or GraphPad Prism v8.

## 3 Results and discussion

### 3.1 GSH metabolism is required for growth of hematopoietic cancer cell lines

To understand the relevance of both GSH-dependent and GSH-independent anti-ferroptosis mechanisms in hematopoietic cancers, we explored the dependency of blood cancer cell lines on *GPX4*, *GCLC*, *GCLM*, *GSS, FSP1* and *SLC7A11 genes*. We selected the dependency score CHRONOS, which is available from the *depmap* portal data [27]. Genes that are required for cell growth will present negative CHRONOS scores, indicating that the inactivation of those genes causes a growth defect. A gene completely essential will generate a CHRONOS score around −1, whereas a non-essential gene will show scores ≥ 0. We detected that cancer cell lines from hematopoietic origin (including leukemia, lymphoma and myeloma cells) present negative CHRONOS scores for genes that code for factors involved in GSH synthesis (*GCLC*, *GCLM* and *GSS*) and for *GPX4* (Fig. 1B). This observation suggests a selective dependency on GSH synthesis and GPX4 for hematopoietic-derived cancer cells. In contrast, *SLC7A11* and *FSP1* presented a selective requirement for lymphoma cell lines, though were not required for growth of leukemia-derived cells (Fig. 1B). To validate these data, we measured the tolerance of a panel of hematopoietic cancer cell lines to the GPX4 inhibitor RSL3 and to the GCL inhibitor L-BSO (Fig. 1C and D and Supp. Fig. 1A and B). All the cell lines tested derived from B- and T-ALL were strikingly sensitivity to RSL3 and L-BSO. On the other hand, the chronic myeloid leukemia (CML) cell line K562 and the colorectal carcinoma cell line HCT-116 were among the most resistant cells to GPX4 and GCL inhibition. Both GPX4 and GSH are key players on preventing ferroptosis, thus suggesting that hematopoietic-derived cancer cells might be more vulnerable to this form of cell death. Indeed, the evaluation of the correlation between gene expression and the tolerance to RSL3 (measured as area under de curve (AUC) in the 823 cancer cell lines from cancer therapeutic response portal) [28] suggests that expression of the anti-ferroptosis factor FSP1 positively correlated with RSL3 AUC (Pearson correlation score of 0.376, Fig. 1E). Thus, cell lines presenting reduced expression of FSP1-dependent anti-ferroptosis defenses might be more prone to undergo ferroptosis upon GPX4 inhibition.

### 3.2 Ferroptosis inducers selectively eliminate ALL cells

**Fig. 2:**
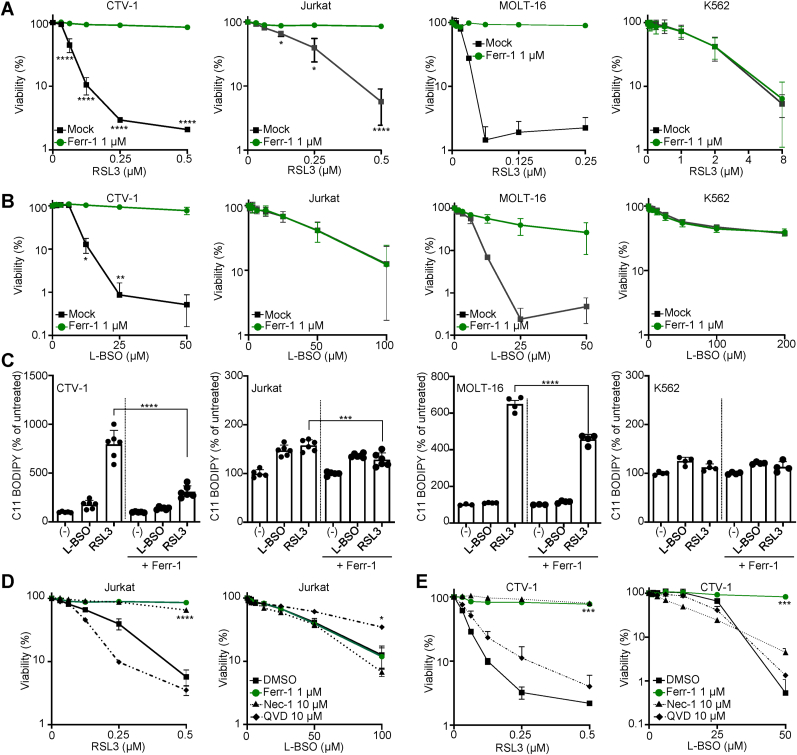
**Ferroptosis underlies GSH metabolism vulnerability in acute lymphoblastic leukemia (ALL). A.** Viability assays performed with the indicated cell lines exposed to increasing concentrations of 1*S*,3*S*-RSL3 (RSL3). Ferrostatin-1 (Ferr-1) at 1 μmol L^−1^ was used as ferroptosis-specific antioxidant. Data are represented as mean ± SD; n = 3 for CTV-1 (6 days); n = 4 for Jurkat (4 days); n = 3 for MOLT-16 (6 days); n = 6 for K562 (4 days). Two-way ANOVA corrected for multiple comparison using a Sidak test. **B.** Viability assays performed with the indicated cell lines exposed to increasing concentrations of l-buthionine-sulfoximine (L-BSO). Ferr-1 was used at 1 μmol L^−1^ in the indicated samples. Data are represented as mean ± SD; n = 3 for CTV-1 (6 days); n = 5 for Jurkat (4 days); n = 3 for MOLT-16 (6 days); n = 6 for K562 (4 days). Two-way ANOVA corrected for multiple comparison using a Sidak test. **C.** Determination of lipid peroxides by C11-BODIPY in cells exposed to RSL3 0.25 μmol L^−1^ or L-BSO 100 μmol L^−1^ for 24 h. The ratio of the geometric mean corresponding to the C11-BODIPY fluorescence intensity calculated for the R-phycoerythrin (PE) channel (Exc. 488 nm/Emm. 585 nm) and for fluorescein isothiocyanate (FITC) channel (Exc. 488 nm/Emm. 530 nm) was calculated and plotted as % of the ratio obtained in the untreated samples (mean ± SD, one-way ANOVA corrected for multiple comparison using a Tukey test, ***P = 0.0001, ****P < 0.0001). **D.** Viability assays performed in Jurkat cells exposed to increasing concentrations of RSL3 or L-BSO and Ferr-1 at 1 μmol L^−1^, necrostatin 1 (Nec-1) 30 μmol L^−1^, or quinoline-Val-Asp-Difluorophenoxymethylketone (QVD) 10 μmol L^−1^ (n = 3, mean ± SD, unpaired *t*-test comparing viability at the highest RSL3 or L-BSO concentration, ****P < 0.0001, *P = 0.0159). **E**. Viability assays performed in CTV-1 cells exposed during 6 days to increasing concentrations of RSL3 or L-BSO and Ferr-1 at 1 μmol L^−1^, Nec-1 30 μmol L^−1^, or QVD 10 μmol L^−1^ (n = 3, mean ± SD, unpaired *t*-test comparing viability at the highest RSL3 or L-BSO concentration, ***P = 0.0001 (RSL3), ***P = 0.0009 (L-BSO)).

GSH is one of the main cellular antioxidants and participates in the detoxification of xenobiotics, conferring resistance to chemotherapeutic drugs and toxins [29]. GSH is also a cofactor for GPX4, which has a more specific role on the detoxification of PLOOHs, suppressing ferroptosis [30]. To address whether the selective sensitivity of ALL cell lines to GPX4 and GSH-synthesis inhibition was because they are more prone to undergo ferroptosis, we determined the tolerance of CTV-1 (T-ALL), Jurkat (T-ALL), MOLT-16 (T-ALL), MOLT-13 (T-ALL), REH (B-ALL), and K562 (CML) cell lines to RSL3 and L-BSO in presence of the ferroptosis antioxidant Ferr-1 (Fig. 2A and B and Supp. Fig. 2A). Remarkably, the sensitivity of ALL cell lines against RSL3 was significantly limited by Ferr-1. In support of this finding, we evaluated the generation of phospholipid hydroperoxides (PLOOH) by the specific dye C11-BODIPY (Fig. 2C). PLOOHs were detected upon exposure to RSL3 in CTV-1, Jurkat and MOLT-16, and only mildly detected in the CML cell line K562. L-BSO also increased PLOOH but to a lesser extent than RSL3. Accordingly, Ferr-1 was able to significantly limit the production of lipid hydroperoxides induced by RSL3 and to less extent L-BSO (Fig. 2C). Interestingly, the toxicity of L-BSO was prevented by Ferr-1 only in some ALL cell lines, consistently with the previous observation in Jurkat cells showing that induction of mitochondrial caspase activation by the Second Mitochondria-Derived Activator of Caspase (SMAC) mimetic compound BV6 is also necessary to induce cell death by GSH-depletion [31,32]. To further assess the cell death mechanism in ALL exposed to GPX4 or GSH-synthesis inhibitors, we evaluated RSL3 and L-BSO toxicity in presence of the caspase inhibitor quinoline-Val-Asp-Difluorophenoxymethylketone (QVD) and of Receptor-interacting serine/threonine-protein kinase 1 (RIPK1) inhibitor Necrostatin-1 (Nec-1). RSL3 tolerance was prevented by Ferr-1 in both Jurkat and CTV-1 cells (Fig. 2D and E). Interestingly, Nec-1 also prevented RSL3 toxicity, in agreement with a recent report showing that Nec-1 prevents ferroptosis independently of RIPK1 [33]. On the other hand, QVD mildly reverted L-BSO toxicity in Jurkat cells, suggesting at least in part cell death is executed by apoptosis in Jurkat cells upon GSH depletion. A plausible mechanism to explain the increased sensitivity of ALL cell lines towards GPX4 inhibition could be that ALL cells sensitive to RSL3 contain less GSH than those cells resistant to this drug. To challenge this hypothesis, we evaluated the level of reduced GSH in Jurkat, CTV-1 and MOLT-16 (all sensitive to RSL3) and in K562 (resistant to RSL3). K562 presented as much reduced GSH as the cell lines sensitive to GPX4 inhibition (Sup. Fig. 2B). The only exception was MOLT-16, which showed a significant less amount of reduced GSH than Jurkat, CTV-1 or K562. Moreover, the exposure of these cell lines to RSL3 did not significantly altered GSH cellular content (Sup. Fig. 2B). In contrast, blocking GCL significantly depleted GSH in both RSL3 sensitive and resistant cells, overall supporting that the selective sensitivity of ALL cells to RSL3 is not caused by differences in the GSH content.

### 3.3 ALL cells lack the expression of FSP1

**Fig. 3:**
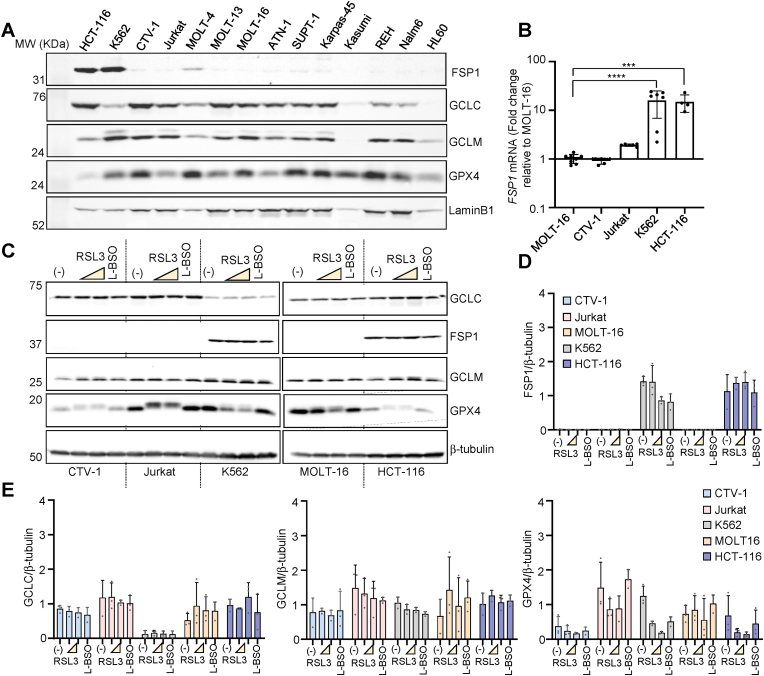
***FSP1* is silenced in acute lymphoblastic leukemia (ALL) cell lines. A.** Immune blot detection of FSP1, GCLC, GCLM, and GPX4 in total protein extracts from the cell lines depicted in the figure. LaminB1 was used as loading control. **B.** RT-qPCR analysis of the expression of *FSP1* in MOLT-16, CTV-1, Jurkat and HCT-116. Data are plotted as expression relative to the level of RNA detected in MOLT-16 (mean ± SD, one-way ANOVA corrected for multiple comparison using a Tukey test, ****P < 0.0001, ***P = 0.0004). **C.** Immune blot detection of FSP1, GCLC, GCLM, and GPX4 in total protein extracts from the cell lines exposed to 1*S*,3*S*-RSL3 (RSL3) or l-buthionine sulfoximine (L-BSO) for 24 h. The concentrations used were 0.25 and 1 μmol L^−1^ RSL3 for CTV-1, Jurkat, K562 and HCT-116. For MOLT-16 RSL3 was used at 0.05 and 0.25 μmol L^−1^. L-BSO was used at 100 μmol L^−1^ in all the cell lines. **D.** Quantification of FSP1 immuneblot shown in C. The data plotted correspond to 3 independent biological replicates. b-tubulin was used as loading control. **E.** Quantification of GCLC, GCLM and GPX4 immuneblots shown in Fig. 3C. The data plotted correspond to 3 independent biological replicates. b-tubulin was used as loading control (mean ± SD; n = 3).

The analysis of the correlation between the tolerance to RSL3 measured as area under de curve (AUC) and gene expression (Fig. 1E) suggests that cells presenting lower expression of *FSP1* are more sensitive to the GPX4 inhibitor RSL3. This observation might indicate that ALL cells present low expression of FSP1. Hence, to evaluate the underlying cause of the increased sensitivity of hematopoietic cancer cells to GSH synthesis and GPX4 inhibition, we analyzed mRNA expression data from all the available cell lines found at depmap. *FSP1* presented a significant low expression in cell lines classified as leukemia (Supp. Fig. 3A). To corroborate these data in hematopoietic cells, we performed western blots in an extended set of leukemia-derived cell lines. GCLC, GCLM and GPX4 were detected in most of the hematopoietic cell lines tested (Fig. 3A), whereas FSP1 was not detected in any of these cell lines but K562 (Fig. 3A and Supp. Fig. 3B). Interestingly, K562 was the only hematopoietic cell line resistant to RSL3 and L-BSO (Fig. 1C and D and Supp. Fig. 1A and 1B). The non-hematopoietic cell line HCT-116 also expressed FSP1 (Fig. 3A), in accordance with previously published data [11]. The expression of FSP1 might be controlled at transcriptional or post-transcriptional level. To gain insights into the regulation of FSP1 in blood cancer cells, we determined the level of *FSP1* mRNA in a subset of cell lines by RT-qPCR. *FSP1* mRNA levels were significantly lower in ALL cells compared to cell lines that show high *FSP1* expression such as K562 and HCT-116 (Fig. 3B). In addition to *FSP1*-lack of expression, the increased sensitivity of ALL cell lines towards ferroptosis inducers might be caused by a differential regulation of GSH biosynthesis genes (*GCLC/GCLM*) or *GPX4*. We addressed the expression of *FSP1*, *GCLC*, *GCLM* and *GPX4* in response to RSL3 or L-BSO in Jurkat, CTV-1, MOLT-16 and K562 by western blot (Fig. 3C). No significant differences in FSP1, GCLC and GCLM protein levels were detected upon 24-h exposure to RSL3 or L-BSO (Fig. 3D and E). We noted a change in the electrophoretic mobility of GPX4 upon exposure to RSL3 likely caused by any of the post-translational modifications described in GPX4 [34], and a net reduction in GPX4 protein level in K562 cells (Fig. 3D and E). Remarkably, K562 -resistant to RSL3- presented the lowest expression of GCLC among the four cell lines, which however did not correlate with the GSH level in this cell line (Supp. Fig. 2B). Overall, the results presented in this section suggest that ALL vulnerability to GPX4 or GSH synthesis inhibition is manly caused by the lack of *FSP1* expression.

### 3.4 FSP1 is hypermethylated in ALL cell lines

**Fig. 4:**
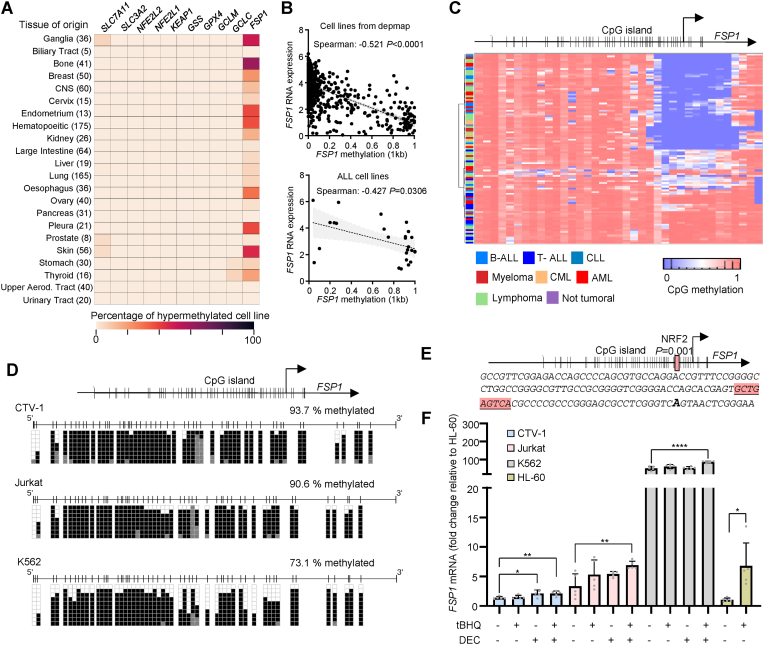
**DNA hypermethylation of *FSP1* is a feature ofacute lymphoblastic leukemia (ALL). A.** Heatmap showing the DNA methylation of *FSP1* promoter in cancer cell lines originated from the tissues described. CNS: Central nervous system. The number in parenthesis represents the number of cell lines included in each group. **B.** Top: Correlation between *FSP1* RNA expression and *FSP1* promoter methylation (1 kb upstream of the transcriptional start site (TSS). Data were obtained from depmap portal. The Spearman correlation and the corresponding *P* value are shown. Bottom: Correlation between *FSP1* RNA expression and *FSP1* promoter methylation (1 kb upstream of the TSS) in acute lymphoblastic leukemia (ALL) cell lines. **C.** DNA methylation analysis by using a Illumina EPIC array in Sanger cell lines from hematopoietic origin. *FSP1* gene structure is shown on the top part of the heatmap highlighting the CpG island found in the promoter region. **D.***FSP1* promoter methylation determined by bisulfite sequencing in CTV-1, Jurkat and K562 cells (sequenced chromosomal region: chr10:71892987 to chr10:71892584, hg19). **E.***FSP1* promoter sequence including the CpG island and the overlapping NRF2-binding site. **F.***FSP1* mRNA expression in CTV-1, Jurkat and K562 cells cultured 8 days in presence 0.5 μmol L^−1^ of 5′-aza-2′-deoxycytidine (decitabine, DEC). Data is plotted relative to the *FSP1* mRNA level detected in HL-60 untreated. For CTV-1 100 nmol L^−1^ of DEC was used. On day 7, tert-butylhydroquinone (tBHQ) was added at 30 mmol L^−1^ (n = 5, mean ± SD, two-tailed unpaired *t*-test, *P = 0.029, **P = 0.0097 (CTV-1), ***P = 0.007 (Jurkat), ****P < 0.0001 (K562), *P = 0.0107 (HL-60).

The striking downregulation of FSP1 in blood-derived cancer cell lines correlated with the increase sensitivity to GPX4 and GCL inhibitors. To address the underlying mechanism of *FSP1* downregulation, we interrogated whether the *FSP1* promoter presents DNA hypermethylation, which might indicate its selective epigenetic silencing. To this end, we mined the Sanger set of human cancer cell lines from different origins comparing the methylation of CpGs found in the promoter region of genes involved in ferroptosis defenses: *SLC7A11, SLC3A2, NFE2L2, NFE2L1, KEAP1, GSS, GCLM, GCLC, GPX4* and *FSP1* [23]. We observed that *FSP1* promoter presents some degree of DNA methylation in most of the cell lines tested (Fig. 4A), suggesting that DNA hypermethylation might impact on *FSP1* expression. Accordingly, a significant inverse correlation could be detected between *FSP1* expression and *FSP1* promoter DNA hypermethylation (Spearman = −0.5210, *P* < 0.0001, Fig. 4B) in the whole panel of cell lines included in the depmap portal [35]. Moreover, this correlation was detected in the subset of ALL cells (Spearman = −0.4247, *P* = 0.0306, Fig. 4B). Overall, these results suggest that the expression of *FSP1* might be at least in part under DNA epigenetic control in ALL-derived cell lines. Indeed, *FSP1* promoter harbors a defined CpG island upstream of its transcriptional start site (TSS) (Fig. 4C). To determine the methylation status of this CpG island, we performed a DNA methylation analysis using the MethylationEPIC BeadChip 850K microarray on multiple hematopoietic cell lines (Fig. 4C, Supp. Fig. 4B). We detected an enrichment of methylated CpGs in the promoter of *FSP1* mainly in ALL, suggesting that DNA methylation controls GSH-independent defenses against ferroptosis in cancer cells from hematopoietic origin. To corroborate this observation, we determined the CpG methylation status of the CpG island detected in *FSP1* promoter by bisulfite sequencing in CTV-1, Jurkat and K562. Interestingly, Jurkat and CTV-1 presented DNA hypermethylation of *FSP1* promoter (>90%), whereas K562 showed partial methylation (73.1%), concordantly with augmented *FSP1* expression detected in this cell line (Fig. 4D). Concordantly, in the hypermethylated T-ALL cell lines Jurkat and CTV-1, a 72-h exposure to the DNA methyltransferase inhibitor decitabine (5′-aza-2′-deoxycytidine, DEC) reduced *FSP1* promotor DNA hypermethylation to 52.5 and 72.5%, respectively (Supp. Fig. 4A). DEC interferes with the activity of DNA methyltransferases reducing the CpG methylation of the DNA [36]. It is still plausible that additional mechanisms might contribute to regulate *FSP1* expression in ALL. Indeed, a recent report indicated that *FSP1* is under the control of nuclear factor erythroid 2-related factor 2 (NFE2L2 or NRF2) in lung cancer [37]. *FSP1* promoter presents a conserved binding site for NRF2 that overlaps the CpG island (Fig. 4E). To evaluate the contribution of NRF2 to *FSP1* regulation in ALL, we determined the *FSP1* mRNA level upon exposure to the NRF2 activator tert-butylhydroquinone (tBHQ). In addition, we used the DNA methyltransferase inhibitor DEC, which interferes with the activity of DNA methyltransferases [36]. The combination of tBHQ and DEC was able to significantly upregulate *FSP1* in Jurkat and CTV-1 cells, which present *FSP1* DNA hypermethylation (Fig. 4F). Interestingly, in HL-60 -an AML cell line that does not present *FSP1* DNA hypermethylation (Supp. Fig. 4B), the exposure to tBHQ was sufficient to upregulated *FSP1* expression. The K562 cell line (CML, partially methylated) presented strong basal expression of *FSP1*. In this model, the combined exposure to tBHQ and DEC increases *FSP1* expression from 52.3 ± 10.5 (untreated) to 88.2 ± 5.2 (tBHQ + DEC) folds compared to HL-60 basal expression (Fig. 4F).

**Fig. 5:**
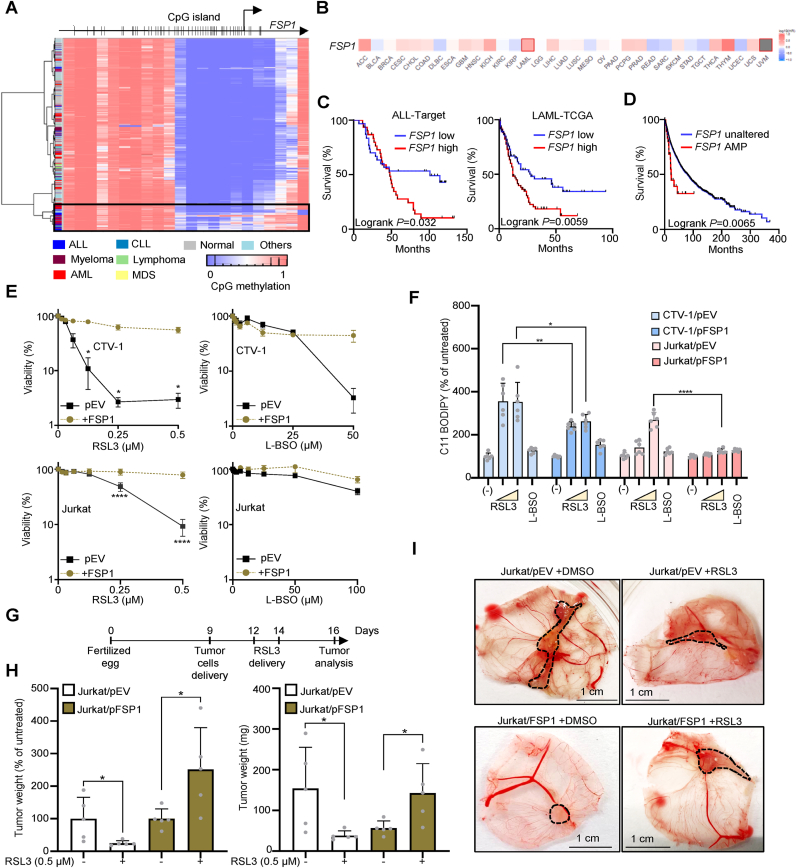
***FSP1* expression confers an advantage to acute lymphoblastic leukemia (ALL) by limiting ferroptosis. A.** Heatmap showing the methylation status of the CpGs found in the promoter of *FSP1* in samples from hematopoietic origin (ALL: T- and B- acute lymphoblastic leukemia; AML: acute myeloid leukemia; CLL: chronic lymphocytic leukemia; MDS: myelodisplastic syndrome). **B.** Survival prognosis analysis according to *FSP1* expression performed in TCGA cohorts. Red and blue tones indicate whether the expression of *FSP1* is a poor or favourable prognostic marker, respectively. Squares indicate the difference between high and low expression groups is significant. Data were calculated using a Mantel–Cox test with p-values corrected for False discovery rate, and the cohort grouped with cut off higher or lower than 50% of the median for the high- and low-*FSP1* expression populations, respectively. **C**. Prognosis analysis in a subset of samples from TARGET project (pediatric ALL). A z-score for the expression of *FSP1* in all the samples was calculated, and samples with z-scores for *FSP*1 > or < than the mean are considered high or low *FSP1*-expressing samples, respectively (TCGA/cBioPortal). **D.** Survival for the pool of TCGA cohorts stratifying samples according to the genomic amplification of *FSP1* (TCGA/cBioPortal). **E.** Viability curves for CTV-1 and Jurkat cell lines stably expressing the protein FSP1. Data are represented as mean ± SD, n = 3. Two-way ANOVA corrected for multiple comparison using a Sidak test. **F.** Determination of lipid peroxides by C11-BODIPY in cells exposed to RSL3 0.25 and 1 μmol L^−1^, or L-BSO 100 μmol L^−1^ for 24 h. The ratio of the geometric mean corresponding to the fluorescence intensity calculated for the R-phycoerythrin (PE) channel (Exc. 488 nm/Emm. 585 nm) and for fluorescein isothiocyanate (FITC) channel (Exc. 488 nm/Emm. 530 nm) was calculated, and plotted as % of the ratio obtained for the untreated sample (mean ± SD, one-way ANOVA corrected for multiple comparison using a Tukey test, **P = 0.0026, *P = 0.0357, ****P < 0.0001). **G.** Scheme depicting the chick embryo chorioallantoic membrane (CAM) tumor assay **H.** Left, tumor weights at day 16 plotted as percentage of the untreated group for the same genotype. Right, net tumor weights at day 16 for Jurkat/pEV and Jurkat/pFSP1 exposed to DMSO or 0.5 mmol L^−1^ (mean ± SD, n = 5, two-tailed unpaired *t*-test)**. I.** Representative pictures of engrafted tumors in CAM at day 16. The dotted line delimitates the tumor. Scale: 1 cm.

### 3.5 FSP1 expression favors cancer progression

To address whether the DNA epigenetic silencing of *FSP1* is a feature of leukemic cells from patients, we investigated the methylation status of the *FSP1* CpG island in cancer biopsies using the Infinium Human Methylation 450 BeadChip (Fig. 5A and Supp. Fig. 5A). A defined subset of primary leukemic cells presented increased *FSP1* DNA methylation, which was not detected in hematopoietic samples from normal donors (Fig. 5A). Accordingly, expression data from cBioPortal show that *FSP1* was barely expressed in leukemia, contrasting with the expression observed in non-hematopoietic cancers (Supp. Fig. 5B). To gain insights into the role of *FSP1* in hematopoietic malignancies in comparison with non-hematopoietic cancers, we performed a prognosis analysis in the 32 TCGA datasets, which include AML (LAML) but not ALL (Fig. 5B) [22]. *FSP1* high expression appeared as a significant worst prognosis marker for overall survival of LAML (Logrank Test *P* value: 0.0059) (Fig. 5C). In the TARGET dataset, the ALL patients that present *FSP1* expression > median also showed a worst overall survival (Logrank Test *P* value: 0.032) (Fig. 5C). Thus, those cancer cells that express *FSP1* might present an advantage leading to a more aggressive cancer evolution. To further support this hypothesis, we look for TCGA tumors in which *FSP1* is amplified. Out of 10967 tumor samples, only 39 show *FSP1* amplification and include patients from adrenocortical carcinoma (ACC), bladder urothelial carcinoma (BLCA), breast invasive carcinoma (BRCA), cholangio carcinoma (CHOL), colon adenocarcinoma (COAD), glioblastoma multiforme (GBM), head and neck squamous cell carcinoma (HNSC), liver hepatocellular carcinoma (LIHC), lung adenocarcinoma (LUAD), ovarian serous cystadenocarcinoma (OV), pancreatic carcinoma (PAAD), prostate adenocarcinoma (PRAD), stomach adenocarcinoma (STAD), and uterine corpus endometrial carcinoma (UCEC). These patients with *FSP1* amplification present a worst prognosis suggesting *FSP1* might function as an oncogene (Fig. 5D) [38]. To address whether *FSP1* confers an advantage to ALL cells in conditions of ferroptosis induction, we generated Jurkat and CTV-1 cells stably expressing FSP1. FSP1-expressing cells were more resistant to ferroptosis induced by GPX4 inhibition (RSL3) and by GSH-synthesis inhibition (L-BSO), overall indicating that the lack of FSP1 expression sensitizes ALL to ferroptosis (Fig. 5E). To further support these findings, we measured PLOOHs in CTV-1 and Jurkat cells overexpressing FSP1 by C11-BODIPY staining. PLOOHs were significantly reduced in both CTV-1 and Jurkat cells exposed to RSL3 (Fig. 5F). In the 24-h exposure protocol, L-BSO was not able to induce significant C11-BODIPY oxidation and, accordingly, no effect on PLOOHs was detected upon overexpression of FSP1 (Fig. 5F). It is likely that longer L-BSO treatment might trigger an accumulation of PLOOHs in CTV-1 cells, though for Jurkat, the cell death mechanism triggered by GSH depletion likely involves caspases and canonical apoptosis (Fig. 2D and E). Finally, to address whether FSP1 also protects ALL cells from ferroptosis during tumor growth, we established a chick embryo chorioallantoic membrane (CAM) model using Jurkat/pEV and Jurkat/pFSP1 (Fig. 5G). This *in vivo* model has long been used in cancer research to evaluate angiogenesis, tumor growth, metastasis, and treatment responses [39,40]. In this assay, cells were deposited in matrigel medium on top of chick embryo membrane (day 9 after fertilization). Three and five days later, RSL3 (or DMSO) at 0.5 μmol L^−1^ was applied in the same place where cells were deposited. At day 16, eggs were opened and tumors weighed. We detected that RSL3 significantly impaired the growth of tumors constituted by Jurkat/pEV. On the other hand, the tumors formed by Jurkat/pFSP1 were not impaired by RSL3 0.5 μmol L^−1^. Moreover, FSP1-overexpressing tumors show a growth advantage in presence of RSL3 (Fig. 5H and I). GPX4-inhibition in conditions of FSP1 overexpression might lead to an increase in reactive oxygen species (ROS), which are not able to kill the cells (because of the presence of FSP1) but can promote growth as previously shown for canonical ROS [41].

## 4 Conclusions

ALL accounts for 26% of all the pediatric cancers including young adults [42]. The most common childhood leukemia is B-ALL, whereas the incidence of T-ALL increases in adults reaching up to 25% of diagnoses leukemia [43]. The overall survival is above 90% in the pediatric population but drops to less than 50% in adults [43]. Identifying genetic features in leukemic cells can guide personalized treatments with optimal outcome. However, conventional chemotherapy is still widely used and usually produces long-term side effects [44], highlighting an urgent need for more selective targets to treat ALL malignancies.

Since the identification of ferroptosis in 2012 [45], intensive work has been carried out to address whether this form of cell death could be induced in cancer but not normal cells [46]. Few advances have been achieved by targeting GSH-dependent axis [47,48], and more recently by using an inhibitor of FSP1 [11,49]. In this work, we identified that ALL tumors lack the expression of the anti-ferroptosis factor FSP1. *FSP1* is under the control of NRF2 in lung cancer [37]. Indeed, *FSP1* promoter contains an NRF2-binding site that overlaps with the CpG island. The DNA methylation of this CpG island at the *FSP1* promoter prevents the expression of *FSP1* in response to NRF2 inducers. In contrast, the non-methylated cell line HL-60 shows upregulation of *FSP1* in response to an NRF2 inducer.

The silencing of *FSP1* in ALL creates a dependency on the anti-ferroptosis axis centered on GPX4, which requires GSH metabolism. We addressed that the ferroptosis inducers RSL3 and L-BSO are significantly more toxic to ALL than to non-hematopoietic cancer cell lines. Moreover, RSL3 impairs tumor growth of Jurkat cells (ALL) but not of the isogenic cells overexpressing FSP1 in a CAM model. Overall, our findings suggest that inducing ferroptosis might be a selective vulnerability with therapeutic impact for ALL.

## Funding

This work was supported by 10.13039/501100011033MCIN/AEI/10.13039/501100011033 and 10.13039/501100000780European Union “NextGenerationEU”/PRTR” Project PCI2021-122045-2B. We thank 10.13039/501100002809CERCA Programme/Generalitat de Catalunya for institutional support. Work at M.E. laboratory is supported by the Health Department PERIS—project no. SLT/002/16/00374 and AGAUR—project no. 2017SGR1080 of the 10.13039/501100002809Catalan Government (Generalitat de Catalunya); Ministerio de Ciencia e Innovación (MCI), 10.13039/501100011033Agencia Estatal de Investigación (AEI) and 10.13039/501100008530European Regional Development Fund (ERDF) project no. RTI2018-094049-B-I00; the Cellex Foundation; and “la Caixa” Banking Foundation (LCF/PR/GN18/51140001). Studies at Roué lab were partially funded by the 10.13039/501100008530ERDF through the 10.13039/100013276Interreg V-A Spain-France-Andorra (POCTEFA) program (EFA360/19, PROTEOblood project). J.C.S was recipient of a Sara Borrell research contract (CD19/00228) from 10.13039/501100004587Instituto de Salud Carlos III. L.B.P. laboratory receives support from 10.13039/501100002923IBioBA-MPSP-CONICET (FOCEM COF 03/11, PICT-PRH 2017–4668), MPI for Metabolism Research (Cologne, Germany) and MPI for Biophysical Chemistry (Gottingen, Germany). A.E.M. is a 10.13039/501100002923CONICET fellow. A.B.C is a fellow of the Spanish Ministry of Education and Vocational Training, under FPU contract no. FPU17/02423. M.E. is an ICREA Research Professor.

## Declaration of competing interest

M.E. is a consultant of Ferrer International and Quimatryx. The authors declare they have no conflict of interest.
