# Supplementary material for: Acute lymphoblastic leukemia necessitates GSH-dependent ferroptosis defenses to overcome FSP1-epigenetic silencing
Source: Redox Biol. 2022 Jul 31;55:102408. doi: 10.1016/j.redox.2022.102408 (PMC9364119; doi:10.1016/j.redox.2022.102408)
Supplement: Multimedia component 1 [file mmc1.pdf]

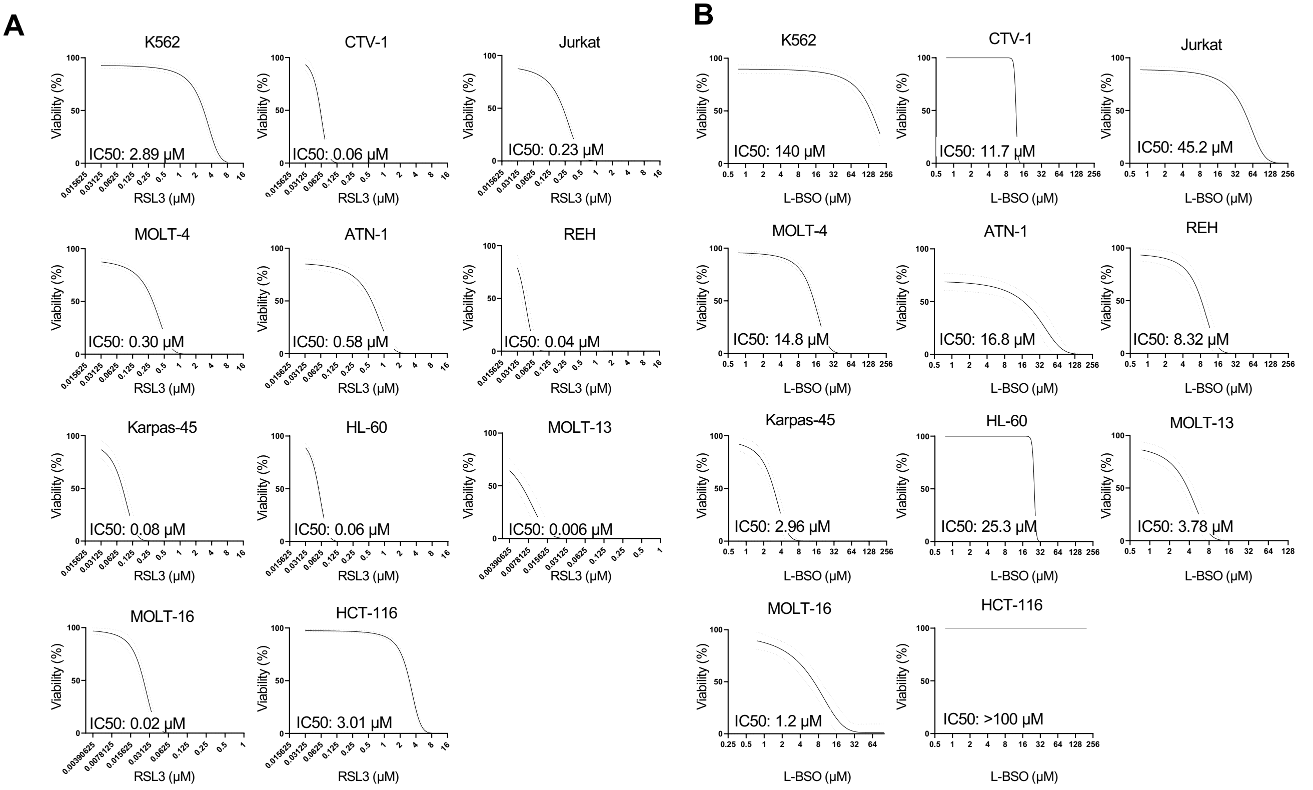

Supplementary Fig 1. Acute lymphoblastic leukemia (ALL) cell lines are sensitive to ferroptosis inducers. A. Viability curves in response to 1*S*,3*S*-RSL3 (RSL3) for the cell lines shown in Fig. 1C. Cells were exposed to RSL3 for 4 (Jurkat, HCT-116, K562, ATN-1) or 6 (MOLT-16, MOLT-13, MOLT-4, REH, Karpas-45, HL-60, CTV-1) days. On the last day, viability was developed by using resazurin. The curves were plotted using Least Square Regression fitting model (GraphPad). The line represents the mean viability relative to the untreated condition at every concentration. The bands with 95% of confidence level are shown in dot lines. The drug concentration that causes a 50% reduction in growth is depicted as IC50. **B.** Data showing doses-response curves for cells exposed to increasing concentrations of L-buthionine sulfoximine (L-BSO). Data were plotted following the approach described in A (n=3 independent experiments per plot). μM represents μmol L^{-1}.

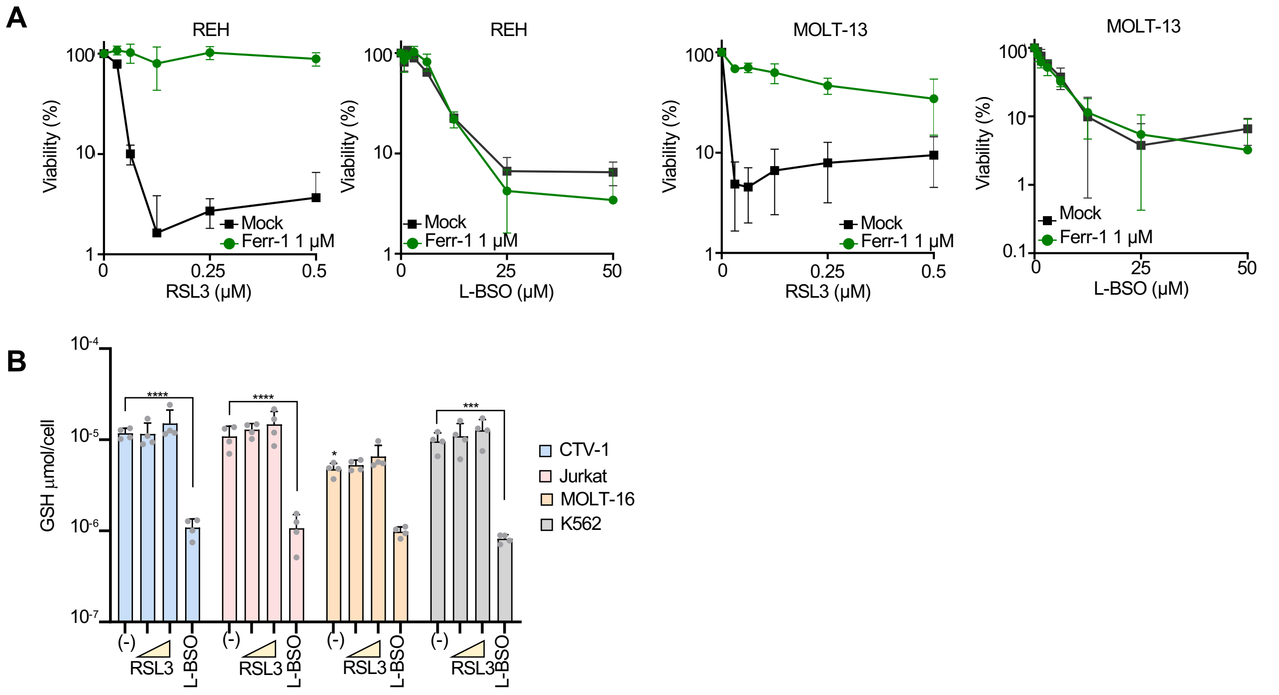

Supplementary Fig. 2. Acute lymphoblastic leukemia (ALL) cell lines are sensitive to ferroptosis inducers. **A**. Viability assays for REH (B-ALL, 6 days) and MOLT-13 (T-ALL, 6 days) in presence of increasing concentrations of (1S,3S)-RSL3 (RSL3) or L-buthionine-sulfoximine (L-BSO) and Ferrostatin-1 (Ferr-1) 1 μmol L^{-1} (μM). Data are represented as mean ± SD, n=3. **B.** Quantification of reduced glutathione (GSH) per cell. RSL3 was used at 0.25 and 1 μmol L^{-1} for CTV-1, Jurkat and K562; and 0.05 and 0.25 μmol L^{-1} for MOLT-16. L-BSO was used at 100 μmol L^{-1} for 24 h (n = 4, mean ± SD, one-way ANOVA corrected for multiple comparison using a Dunnet test, ****P*=0.0005, *****P*<0.0001). μM (μmol L^{-1}).

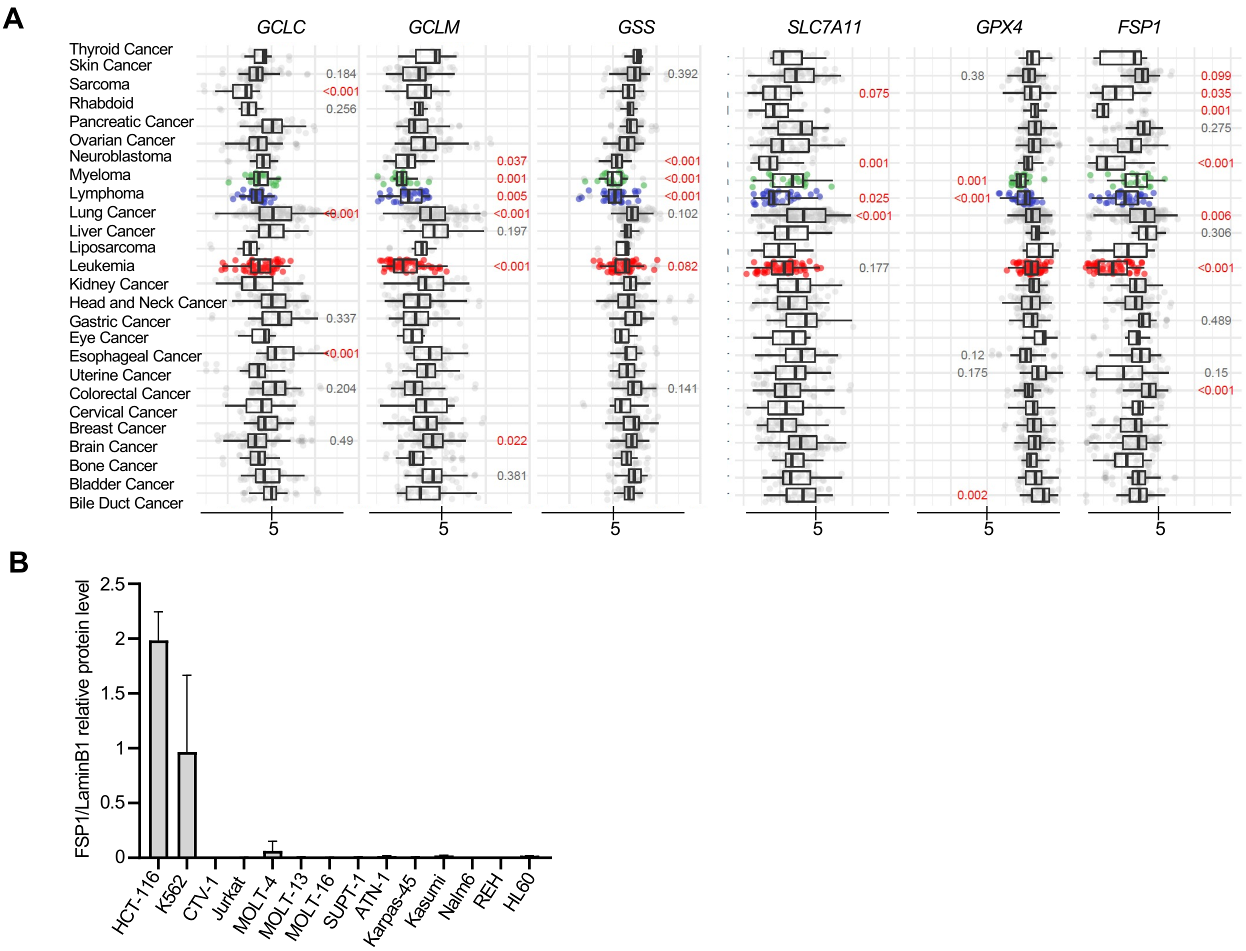

Supplementary Fig. 3: *FSP1* is silenced in acute lymphoblastic leukemia (ALL) cell lines. A. *FSP1* mRNA expression (log2(TPM+1) Expression 22Q2 Public) for cell lines grouped according to their tissue origin. Data were obtained from depmap portal, the x-axis scale goes from 0 to10. The statistical analysis was performed applying a one-way ANOVA and a Tukey test for multiple comparison in an R environment. The statistical significance is shown for the comparison of the selected sample against the mean *FSP1* expression in the entire pool of samples. **B.** Quantification of FSP1 immune blots shown in Fig. 3A (mean ± SD; n=2).

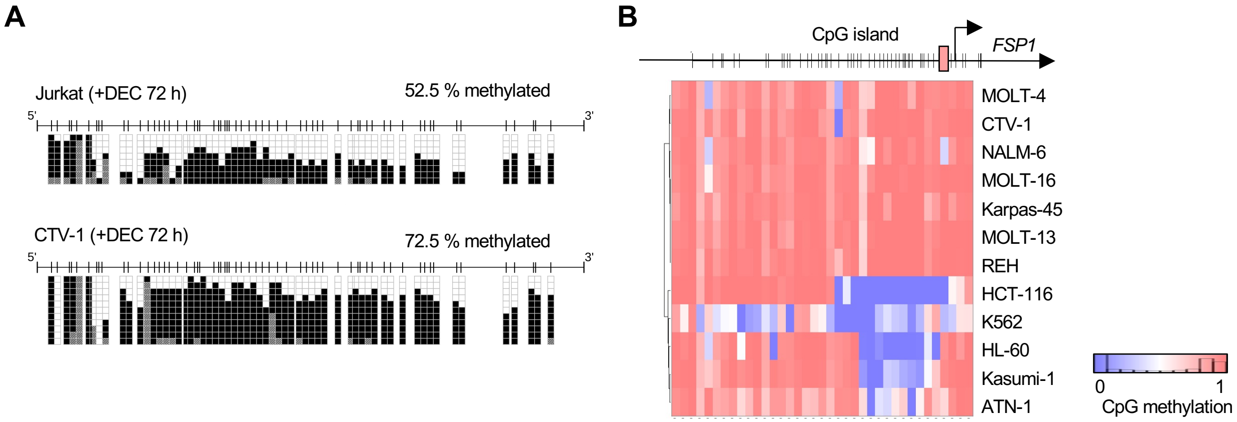

Supplementary Fig. 4. DNA hypermethylation of *FSP1* is a feature of acute lymphoblastic leukemia (ALL). A. *FSP1* promoter methylation determined by bisulfite sequencing in Jurkat and CTV-1 cells exposed to 1 μmol L^{-1} 5'-aza-2'-deoxycytidine (decitabine, DEC) for 72 h (sequenced chromosomal region: chr10:71892987 to chr10:71892584, hg19). **B.** Heatmap showing the CpG methylation of *FSP1* promoter in a subset of cell lines selected from Fig. 4C.

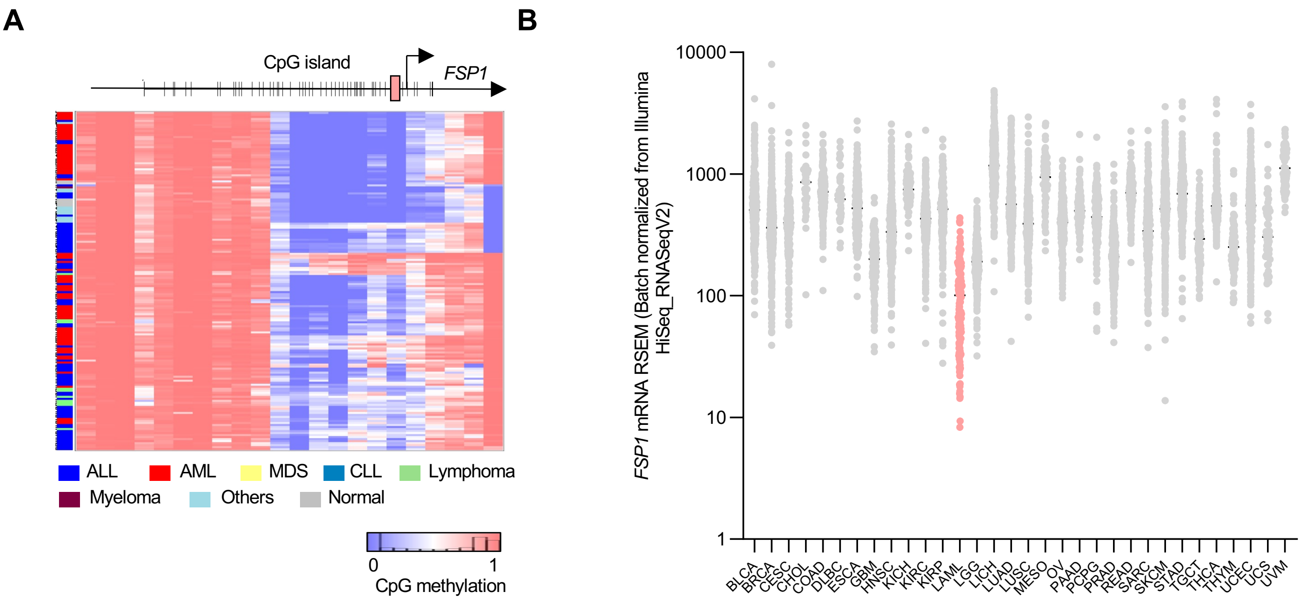

Supplementary Fig. 5. *FSP1* expression is reduced in leukemia samples. A. Zommed in area (black box in Fig. 5A) of the heatmap showing the DNA methylation status of *FSP1* promotor in patient samples with hematological malignancies. ALL: Acute lymphoblastic leukemia; AML: Acute myeloid leukemia; MDS: Myelodysplastic syndrome; CLL: Chronic lymphocytic leukemia. The CpG methylation goes from 0 (blue) to 1 (red). **B.** *FSP1* mRNA expression in the whole panel of TCGA tumors. Data were obtained from cBioPortal (RNASeq V2 data from TCGA normalized using RSEM). Leukemia samples show significant differences with all the cancer but encapsulated glioma, glioblastoma, diffuse glioma and miscellaneous neuroepithelial tumor (one-way ANOVA, $P<0.05$). BLCA: Bladder Urothelial Carcinoma, BRCA: Breast invasive carcinoma, CESC: Cervical squamous cell carcinoma and endocervical adenocarcinoma, CHOL: Cholangio carcinoma, COAD: Colon adenocarcinoma, DLBC: Lymphoid Neoplasm Diffuse Large B-cell Lymphoma, ESCA: Esophageal carcinoma, GBM: Glioblastoma multiforme, HNSC: Head and Neck squamous cell carcinoma, KICH: Kidney Chromophobe, KIRC: Kidney renal clear cell carcinoma, KIRP: Kidney renal papillary cell carcinoma, LAML: Acute Myeloid Leukemia, LGG: Brain Lower Grade Glioma, LIHC: Liver hepatocellular carcinoma, LUAD: Lung adenocarcinoma, LUSC: Lung squamous cell carcinoma, MESO: Mesothelioma, OV: Ovarian serous cystadenocarcinoma, PAAD: Pancreatic adenocarcinoma, PCPG: Pheochromocytoma and Paraganglioma, PRAD: Prostate adenocarcinoma, READ: Rectum adenocarcinoma, SARC: Sarcoma, SKCM: Skin Cutaneous Melanoma, STAD: Stomach adenocarcinoma, TGCT: Testicular Germ Cell Tumors, THCA: Thyroid carcinoma, THYM: Thymoma, UCEC: Uterine Corpus Endometrial Carcinoma, UCS: Uterine Carcinosarcoma, UVM: Uveal Melanoma
